# Effect of Encapsulated Beet Extracts (*Beta vulgaris*) Added to Yogurt on the Physicochemical Characteristics and Antioxidant Activity

**DOI:** 10.3390/molecules26164768

**Published:** 2021-08-06

**Authors:** Martha A. Flores-Mancha, Martha G. Ruíz-Gutiérrez, Rogelio Sánchez-Vega, Eduardo Santellano-Estrada, América Chávez-Martínez

**Affiliations:** 1 Departamento de Tecnología de Productos de Origen Animal, Facultad de Zootecnia y Ecología, Universidad Autónoma de Chihuahua, Periférico Francisco R. Almada km 1, Chihuahua 31000, CI, Mexico; 99azu.flores@gmail.com (M.A.F.-M.); rsanchezv@uach.mx (R.S.-V.); esantellano@uach.mx (E.S.-E.); 2 Departamento de Investigación y Posgrado, Facultad de Ciencias Químicas, Universidad Autónoma de Chihuahua, Circuito Universitario s/n Campus Universitario 2, Chihuahua 31125, CI, Mexico; mruizg@uach.mx

**Keywords:** yogurt, betalains, encapsulation, lyophilization, antioxidant activity, polyphenols

## Abstract

Beet has been used as an ingredient for functional foods due to its high antioxidant activity, thanks to the betalains it contains. The effects of the addition of beet extract (liquid and lyophilized) on the physicochemical characteristics, color, antioxidant activity (AA), total betalains (TB), total polyphenols (TP), and total protein concentration (TPC) were evaluated on stirred yogurt. The treatments (T1-yogurt natural, T2-yogurt added with beet juice, T3-added extract of beet encapsulated with maltodextrin, and T4-yogurt added with extract of beet encapsulated with inulin) exhibited results with significant differences (*p* < 0.05). The highest TB content was observed in T2 (209.49 ± 14.91), followed by T3 (18.65 ± 1.01) and later T4 (12.96 ± 0.55). The highest AA was observed on T2 after 14 days (ABTS˙ 0.819 mM TE/100 g and DPPH˙ 0.343 mM TE/100 g), and the lowest was found on T1 at day 14 (ABTS˙ 0.526 mM TE/100 g and DPPH˙ 0.094 mM TE/100 g). A high content of TP was observed (7.13 to 9.79 mg GAE/g). The TPC varied between 11.38 to 12.56 µg/mL. The addition of beet extract significantly increased AA in yogurt, betalains being the main compounds responsible for that bioactivity.

## 1. Introduction

Dairy derivatives are the main segment among functional foods, representing around 74% of all functional products [1].Yogurt is a functional dairy food that is obtained from the fermentation of milk with lactic acid bacteria (LAB); it is consumed around the world and is traditionally recognized as a healthy food due to its own characteristics [2], which can be improved by incorporating natural ingredients such as fruit extracts [3], tea [4], olive leaf extract [5], paprika juice [6], coriander and cumin seeds [7], etc. It has been reported that the high oxidative stability of yogurt is due to the peptides released during the fermentation carried out by LAB [8]. According to the latest studies, the use of various additives and fruit, in yogurt production, has a significant influence its quality [9,10]. Thus, the addition of natural food materials increases the nutritive value of yogurt. The fortification of yogurt using different bioactive compounds has been studied by different authors [4,5,6,7,11,12]; encapsulation by extrusion can significantly extend the stability of natural β-carotene with potential use as a functional ingredient in yogurt [13]. The addition of hibiscus flower essential oil can also be used to increase the antioxidant capacity of yogurt due to anthocyanin content [12]. Green, white and black tea can be used successfully to enhance the antioxidant properties of yogurt and provide sustained antioxidants during storage [4]. On the other hand, the addition of carotenoids in a fermented-maize product, similar to yogurt, can induce a cholesterol lowering effect [14].

Beet (*Beta vulgaris* sp.) represent the main commercial source of betalains [15,16,17], in addition to containing polyphenols, both being powerful antioxidants. In recent decades, the functional properties that these compounds confer on health have been studied, among which their antioxidant (AA), antidiabetic, anti-inflammatory and anticancer activity stand out [18,19,20,21,22,23,24,25]. Beet extract (betanin) is an approved color under the code 73.40 by the US Food and Drug Administration [17,26] and by the European Union designated with the number E162 [22,27]. Betalains and polyphenols are unstable in the presence of light [28], high temperatures [29], alkaline pH [30,31], enzymatic activity [32], and presence of oxygen and/or metals [33,34]. Due to their low stability, their use in food has been restricted [3,35,36,37,38,39,40,41,42]. However, it has been shown that the encapsulation of betalains and polyphenols in different edible matrices (maltodextrin and inulin) can increase the stability of these compounds and therefore retain their antioxidant and anti-radical activities [21,43,44,45,46,47,48,49]. Therefore, the objective of this work was to evaluate the effect of adding beet extract (*Beta vulgaris* sp.) encapsulated with different carrier agents on the physicochemical characteristics, color, betalain and polyphenol content, and antioxidant capacity of yogurt during shelf life. The treatments were as follows: T1-natural yogurt as control, T2-yogurt added with beet juice, T3-added extract of beet encapsulated with maltodextrin, and T4-yogurt added with extract of beet encapsulated with inulin.

## 2. Results and Discussion

### 2.1. Physicochemical Analysis

The treatments added with encapsulated beet extract (T3 and T4) presented higher protein content (4.88 ± 0.22 and 4.84 ± 0.48, respectively), showing a significant difference (*p* < 0.05), compared to T1 (3.87 ± 0.31) and with T2 (3.88 ± 0.36) (*p* < 0.05). Regarding the percentage of fat, treatments T3 (5.49 ± 0.81) and T4 (5.36 ± 0.32) had a higher content in contrast to T1 (5.09 ± 0.45) and T2 (5.02 ± 0.86) (Table 1), although in this determination, there was no significant difference (*p* > 0.05) between the treatments. Other studies indicate that yogurts added with rubas (*Ullucus tuberosus*) concentrate, curuba (*Passiflora mollissima* Bailey) extract, chocolate, and oats also showed a significant increase in protein and fat content [50,51,52,53]. The moisture percentage showed a significant difference (*p* < 0.05) in the four treatments, this behavior could be attributed to the fact that the beet extracts were in different forms (juice or powders), since in treatments 3 and 4 added with powders, the moisture content was lower (78.10 ± 0.17 and 78.60 ± 0.33) than in T1 (78.94 ± 0.15), likewise, the moisture percentage of T2 was higher (79.92 ± 0.12) than T1. Regarding the percentage of ash, there was no significant difference (*p* > 0.05) between the treatments.

### 2.2. pH and Syneresis (SYN)

**Figure 1:**
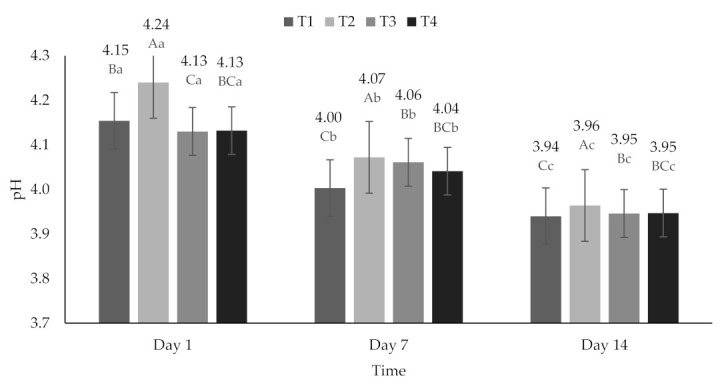
Hydrogen potential (pH) of yogurt added with beet extracts. T1 = Natural yogurt (Y), T2 = Y added with beet juice, T3 = Y added with extract encapsulated with Maltodextrin, and T4 = Y added with extract encapsulated with Inulin. ^A,B,C^ Values with different superscript indicate significant statistical difference between treatments (*p* < 0.05). ^a,b,c^ Values with a different superscript indicate a statistically significant difference over time (*p* < 0.05).

**Figure 2:**
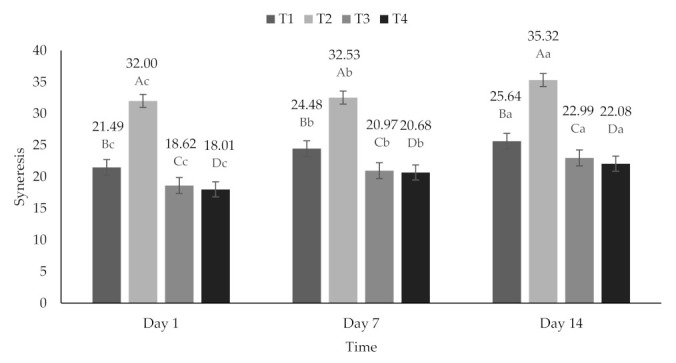
Percentage of syneresis of yogurt added with beet extracts. T1 = Natural yogurt (Y), T2 = Y added with beet juice, T3 = Y added with extract encapsulated with Maltodextrin, and T4 = Y added with extract encapsulated with Inulin. ^A,B,C,D^ Values with different superscript indicate significant statistical difference between treatments (*p* < 0.05). ^a,b,c^ Values with different superscript indicate statistical difference significant over time (*p* < 0.05).

All treatments presented a decrease in pH over time (*p* < 0.05). These values were 4.13 ± 0.052 on day 1, 4.04 ± 0.030 on day 7, and 3.95 ± 0.010 on day 14 (Figure 1), and that decrease in pH could be attributed to the microbial activity of the lactic acid bacteria present in yogurt [52,54]. The degree of SYN varied from 18.01 to 35.32%, presenting significant differences (*p* < 0.05) between treatments and days of storage (Figure 2).

These differences between treatments could be attributed to the fact that the components of the powders, specifically maltodextrin and inulin, favored water retention, because they contribute to the mesh effect in the three-dimensional network of the gel formed in the yogurt [55]. In addition, syneresis increased in all treatments during storage, possibly due to the loss of stability of the yogurt components [56]. This behavior is also due to the decrease in pH during storage, since it can have a contraction effect in the casein micelle matrix causing greater elimination of whey [57].

### 2.3. Color

The values of the color parameters L*, a* and b* can be observed in Table 2. The values of L* decreased significantly for T1, T3, and T4, although this value increased in T2 from 43.70 at day 1 to 50.88 at day 14 (*p* < 0.05). In relation to the parameter a* (Table 2), this was reduced in T3 after storage, and this trend was reported [58] in microcapsules of beets using M as a vehicle (day 0 to a* = 8.39, day 7 to a* = 1.71); however, the a* values for T1, T2, and T4 showed an increase over time (*p* < 0.05). In general, the b* values also showed an increase over time (*p* < 0.05). The degradation of BC leads to the formation of compounds with a yellow color and, this is reflected in the increase in the b* parameter [38]. Color saturation (Chroma) and hue (Hue angle) indicated differences (*p* < 0.05) between treatments and over time; however, treatments added with beet powder (T3 and T4) did not show differences (*p* > 0.05). The color change (∆E) between treatments and days was also different (*p* < 0.05).

### 2.4. Antioxidant Activity (AA)

**Figure 3:**
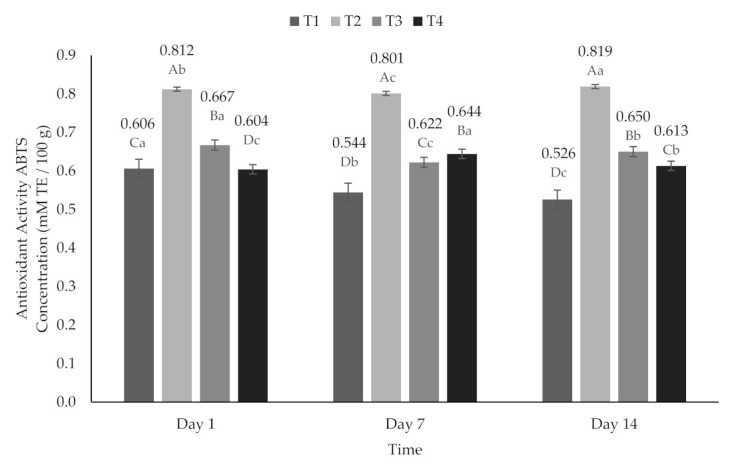
Antioxidant activity (ABTS˙ method) of yogurt added with beet extract. T1 = Natural yogurt (Y), T2 = Y added with beet juice, T3 = Y added with extract encapsulated with Maltodextrin, and T4 = Y added with extract encapsulated with Inulin. ^A,B,C,D^ Values with different superscript indicate significant statistical difference between treatments (*p* < 0.05). ^a,b,c^ Values with a different superscript indicate significant statistical difference over time (*p* < 0.05).

**Figure 4:**
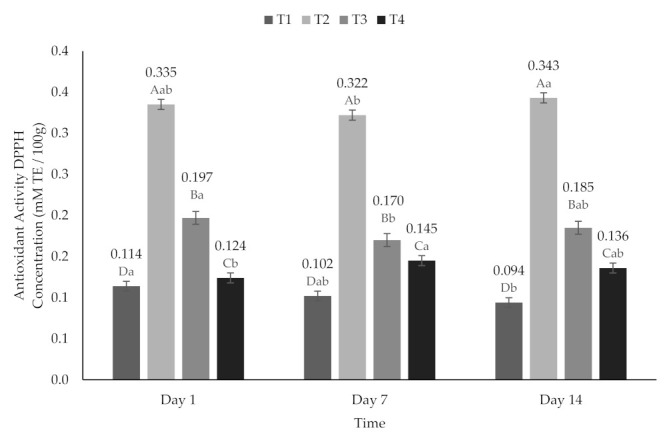
Antioxidant activity (DPPH˙ method) of yogurt added with beet extract. T1 = Natural yogurt (Y), T2 = Y added with beet juice, T3 = Y added with extract encapsulated with Maltodextrin, and T4 = Y added with extract encapsulated with Inulin. ^A,B,C,D^ Values with different superscript indicate significant statistical difference between treatments (*p* < 0.05). ^a,b^ Values with a different superscript indicate significant statistical difference over time (*p* < 0.05).

Statistically significant differences (*p* < 0.05) were found for the AA variable between the treatments (*p* < 0.05) in ABTS˙ (Figure 3) and DPPH˙ (Figure 4). The highest AA was observed on T2 after 14 days (ABTS˙ 0.819 mM TE/100 g and DPPH˙ 0.343 mM TE/100 g), and the lowest AA was found on T1 at day 14 (ABTS˙ 0.526 mM TE/100 g and DPPH˙ 0.094 mM TE/100 g). Among the treatments added with encapsulated extract, the highest AA was found in T3 on day 1 (ABTS˙ 0.667 mM TE/100 g and DPPH˙ 0.197 mM TE/100 g). On the other hand, T4 presented higher AA on day 7 (ABTS˙ 0.644 mM TE/100 g and DPPH˙ 0.145 mM TE/100 g); it is important to highlight that for this treatment, the AA was higher on day 14 (ABTS˙ 0.613 mM TE/100 g and DPPH˙ 0.136 mM TE/100 g) even than on day 1 (ABTS˙ 0.604 mM TE/100 g and DPPH˙ 0.124 mM TE/100 g) (Figure 4). Likewise, a decrease on AA was reported previously, followed by a 10 day stability period and an increase in AA by day 14 [59]. This behavior could be due to compounds with high antioxidant activity that were formed or released during storage due to the adsorption of water by encapsulation or to the interaction of some components in the stored sample with oxygen or other components of the sample [60]. In addition, it has been reported that a longer storage time and a greater water activity produce greater antioxidant activity to extinguish radicals [59,61].

### 2.5. Total Polyphenols (TP)

**Figure 5:**
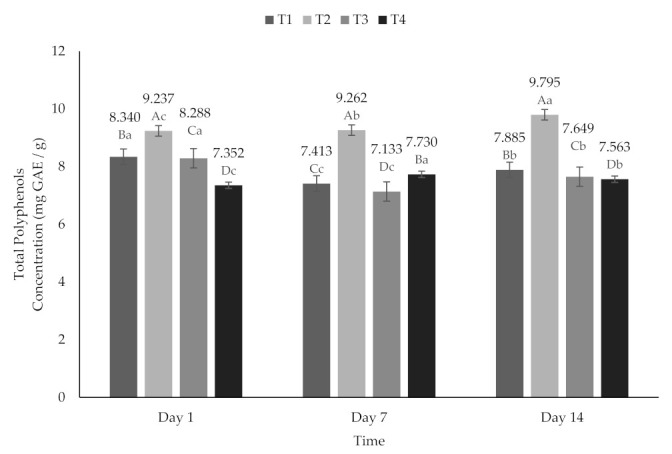
Total polyphenol content of yogurt added with beet extract. T1 = Natural yogurt (Y), T2 = Y added with beet juice, T3 = Y added with extract encapsulated with Maltodextrin, and T4 = Y added with extract encapsulated with Inulin. ^A,B,C,D^ Values with different superscript indicate significant statistical difference between treatments (*p* < 0.05). ^a,b,c^ Values with a different superscript indicate significant statistical difference over time (*p* < 0.05).

A high content of TP was observed from 7.13 to 9.79 mg of GAE/g, (Figure 5) referring to that reported previously 4.88 mg of GAE/g [58]. In cactus pear (*Opuntia ficus* indica) powders values of 14.67 mg of GAE/g have been reported [62]. The concentration of TP showed a behavior reported previously [48], where report recoveries and polyphenol formation of more than 100% with respect to the initial time, as a consequence of the hydrolysis of the beet polyphenol conjugates during storage [48]. Storage with high water activity facilitates oxidation reactions, causing phenolic compounds to react with oxygen and produce an enriched medium that has strong radical elimination and reduction properties [59]; it is worth mentioning that these studies were carried out only on powders.

### 2.6. Total Betalains (TB)

**Figure 6:**
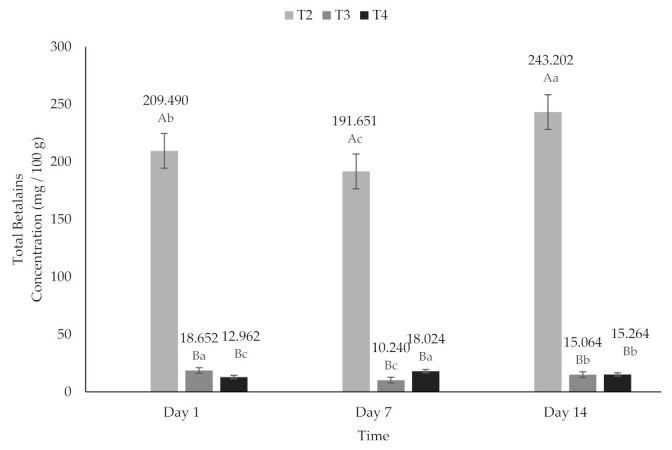
Total betalain content of yogurt added with beet extract. T2 = Y added with beet juice, T3 = Y added with extract encapsulated with Maltodextrin, and T4 = Y added with extract encapsulated with Inulin. ^A,B^ Values with different superscript indicate significant statistical difference between treatments (*p* < 0.05). ^a,b,c^ Values with a different superscript indicate significant statistical difference over time (*p* < 0.05).

Several investigations have been carried out on the use of encapsulating agents to prevent the degradation of betalains [35,58,63,64]; however, the most of the stability studies were conducted on powder samples. In the present study, the powder was added to a non-Newtonian fluid. The stability of the extracts was evaluated in the yogurts in terms of TB content (Figure 6), during 14 days of storage at 4 °C and in absence of light. The highest pigment content was observed in T2 after 14 days (243.20 mg/100 g); statistically significant differences (*p* < 0.05) in TB content were observed after 7 (191.65 mg/100 g) and 14 days of storage compared to its content on day 1 (209.40 mg/100 g). The TB content increased with the fermentation time, and that event could be due to acid hydrolysis and the bioconversion of condensed phenols [29,65]. In addition, during fermentation, microbial activity caused an intense release of betalains responsible for the increase in TB content [61]. Of the treatments added with encapsulated extract (T3 and T4), the highest TB content was found in T3 on day 1 (18,652 mg/100 g), and the highest TB content for T4 was on day 7 (18,024 mg/100 g). However, while T3 showed a decrease in TB on day 7 (10.24 mg/100 g) and subsequently an increase on day 14 (15.06 mg/100 g), T4 had an increase in TB on day 7 (18.02 mg/100 g) and on day 14 a decrease (15.26 mg/100 g). However, it is important to note that on day 14 the quantification of TB in T4 was higher, even than on day 1 (12.96 mg/100 g) (Figure 6).

Encapsulation with maltodextrin was effective in stabilizing betalains after 14 days of storage [66]. Unlike what was reported by Omae et al. [46], in this study, the addition of inulin did increase the stability of BC during storage, and a linear relationship was not observed from the graphs of TB content versus time. According to previous studies [28,29,67,68], the degradation of BC is the result of a hydrolysis reaction that produces cyclo-DOPA-D-glucoside (CDG) and AB; this degradation reaction is reversible, and involves a condensation of the Schiff’s base of the CDG amine with the aldehyde of betalamic acid (BA) [69]. On the other hand, the degradation of betalamic pigments derived from beets was reported [46,58,59,63].

**Figure 7:**
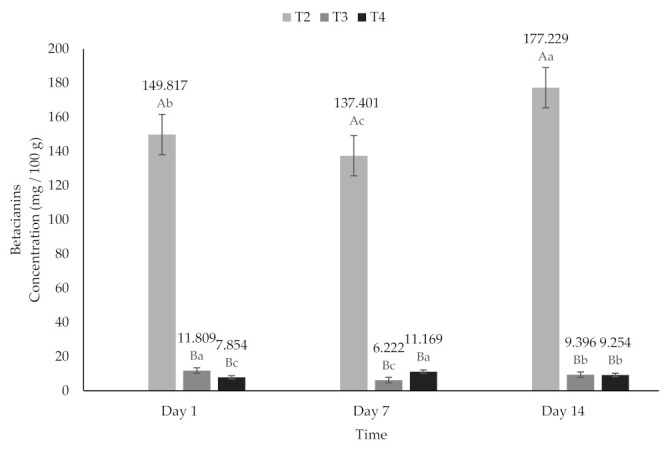
Betacyanins content of yogurt added with beet extract. T2 = Y added with beet juice, T3 = Y added with extract encapsulated with Maltodextrin, and T4 = Y added with extract encapsulated with Inulin. ^A,B^ Values with different superscript indicate significant statistical difference between treatments (*p* < 0.05). ^a,b,c^ Values with a different superscript indicate significant statistical difference over time (*p* < 0.05).

**Figure 8:**
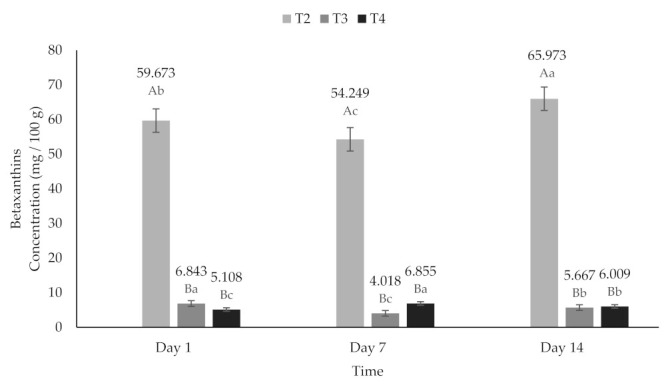
Betaxanthins content of yogurt added with beet extract. T2 = Y added with beet juice, T3 = Y added with extract encapsulated with Maltodextrin, and T4 = Y added with extract encapsulated with Inulin. ^A,B^ Values with different superscript indicate significant statistical difference between treatments (*p* < 0.05). ^a,b,c^ Values with a different superscript indicate significant statistical difference over time (*p* < 0.05).

Beets have been reported to have higher levels of betacianins (BC) than betaxanthins (BX) [63,70,71,72]. According to the above, in this investigation, a higher content of BC was found compared to BX. The contents of BC (6.22 to 11.809 mg/100 g) and BX (4.01 to 6.85 mg/100 g) (Figure 7 and Figure 8) in the treatments added with encapsulated beet extract (T3 and T4) in this study were lower than those reported by Janiszewska [63] and higher than those reported by Ravichandran et al. [72] (118.0 mg/100 g and 3.5 mg/100 g, respectively).

### 2.7. Total Protein Concentration (TPC)

**Figure 9:**
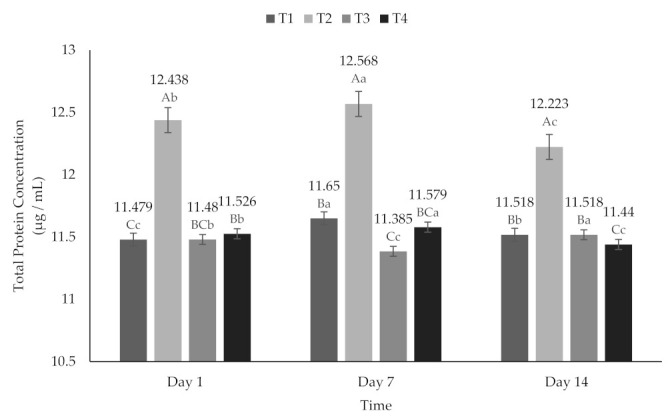
Total protein concentration of yogurt added with beet extract. T1 = Natural yogurt (Y), T2 = Y added with beet juice, T3 = Y added with extract encapsulated with Maltodextrin, and T4 = Y added with extract encapsulated with Inulin. ^A,B,C^ Values with different superscript indicate significant statistical difference between treatments (*p* < 0.05). ^a,b,c^ Values with a different superscript indicate significant statistical difference over time (*p* < 0.05).

The TPC (Figure 9) ranged from 11.385 to 12.568 µg/mL. However, they had a statistically significant difference (*p* < 0.05) between treatments and over time. This behavior is probably due to the extracellular proteins secreted by bacteria during the fermentation time [73]. Furthermore, the increase in the total content of betalains has a positive correlation regarding the total concentration of proteins, due to the fact that they are nitrogenous compounds [74].

### 2.8. Correlation between Variables

Table 3 shows the correlation coefficient (r) and level of significance between the response variables of the yogurts. A strong negative correlation (r = −0.9410, *p* < 0.0001) was found between L* and TB and a positive correlation (r = 0.7054, *p* < 0.0001) between the value of a* and TB. This indicates that the higher the TB content, the lower the luminosity and the higher the concentration of red color. These correlations have already been reported in prickly pear pulp micro particles [48,75,76] and in prickly pear pulp coloring powders [44]. Like Pitalua et al. [59], observed a strong positive correlation (r = 0.8615, *p* < 0.0001) between TP and TB. It is important to highlight that betalains are the main polyphenols present in beets [58]; therefore, a positive association between both variables was expected. In addition, positive correlations were obtained (r = 0.9994, *p* < 0.0001), (r = 0.9960, *p* < 0.0001) between TB and BC and between TB and BX, respectively. On the other hand, a high positive correlation (r = 0.7946, *p* < 0.0001) was found between TB and AA, which could be due to the presence of betalamic acid, since it acts as an antioxidant agent. However, if the acid molecule does not have hydroxyl groups, betalamic acid does not have antioxidant capacity [64]. Likewise, a positive correlation was found (ABTS˙ r = 0.8263 and DPPH˙ r = 0.8002, *p* < 0.0001) between AA and TP. The increase in AA during storage can occur because in these periods, new phenolic compounds capable of extinguishing radicals are released or formed [77]. A positive correlation was found between TPC and AA (ABTS˙ r = 0.8022 and DPPH˙ r = 0.8126, *p* < 0.0001) and TP (r = 0.7917, *p* < 0.0001) and TB (r = 0.8822, *p* < 0.0001). These correlations could be to compounds with high AA that were formed or released during storage, thanks to the adsorption of water [60], and it can be observed despite the presence of betalains [64]. Storage with high water activity facilitates hydrolysis, causing phenolic compounds to react with oxygen and produce an enriched medium that has strong radical elimination and reduction properties [65,73].

## 3. Materials and Methods

### 3.1. Materials

Between March and July 2018, beet, whole milk (LALA^®^, Gomez Palacio, Dgo., Mexico), whole cream (LALA^®^, Gomez Palacio, Dgo., Mexico), powdered milk (NIDO^®^, Ocotlan, Mex., Mexico), table sugar, and natural yogurt (LALA^®^, Gomez Palacio, Dgo., Mexico) were purchased in a local market in Chihuahua, Mexico. Maltodextrin (DE 10) and agave inulin were purchased from Sigma-Aldrich (St. Louis, MO, USA).

### 3.2. Reagents

All other reagents used were analytical grade. Reagents 2,2′-azino-bis (3-ethylbenzothiazoline-6-sulfonic acid) (ABTS), 2,2-diphenyl-1-picrylhydrazyl (DPPH), Trolox, ammonium salt, potassium persulfate, Folin–Ciocalteu, sodium carbonate, gallic acid, trichloroacetic acid, citrate, and phosphate standard reagent were analytical grade and obtained from Sigma-Aldrich (St. Louis, MO, USA). J. T. Baker provided high-performance liquid chromatography (HPLC)-grade methanol and HPLC-grade water (Mexico City, Mexico). A deionizer was used to obtain deionized water (Barnstead, Thermo Scientific, Waltham, MA, USA).

### 3.3. Yogurt Elaboration

To elaborate yogurts, milk was heated to 95 °C for five minutes, then cooled to 45 °C, and then, powdered milk (8%), cream (3%), sugar (3%), and the inoculum (10%) were added. Next, yogurts were incubated ((Thermo Fisher Scientific, Model 3721, USA) at 42 °C for approximately 4:30 h, until reaching a pH of 4.2. Once this acidity was reached, the yogurt was shaken and stored at 4 °C for 12 h. Subsequently, the beet extracts (liquid or lyophilized) were added to the yogurt; a shake was made, and finally, these were stored at 4 °C for 14 days.

### 3.4. Preparation of Beet Extracts

A household extractor (Cold Press 900W, Breville, Sydney, Australia) was used to extract the juice from the beets. The encapsulating agents (Maltodextrin-M and Inulin-I) were added to the red beet juice at room temperature at an amount of 30 g of dry matter per 100 mL, as described by Antigo et al. [58]. Next, the mixtures were homogenized (10 min) with a dispersion tool (Vortex-Ultra-Turrax IKA T18 basic) (S18N-19G, IKA Works Inc., Wilmington, NC, USA) and frozen for 48 h at 20 °C previously to freeze-dried (4 days at −85 °C and 0.035 mbar pressure) in a Labconco Niro Mobile Minor Freeze-dryer. Finally, pure beet (B), beet extract encapsulated with maltodextrin (M), and beet extract encapsulated with inulin (I) were kept at 9 ± 3 °C for further analysis. The characterization of these extracts has been reported previously [42].

### 3.5. Treatments

Four treatments with three repetitions were elaborated under a completely random statistical model. The description of the treatments was based on the addition of beet extracts (liquid or lyophilized and encapsulated). Control treatment was yogurt without the addition of beet extract (T1), yogurt added with 100 mL of liquid beet extract per liter (T2), yogurt added with 600 mg of beet extract encapsulated with maltodextrin per liter (T3), and yogurt added with 600 mg of beet extract encapsulated with inulin per liter (T4). Treatments were kept in refrigeration (4 °C) for 14 days, taking samples on days 1, 7, and 14 to evaluate color, pH, syneresis, betalains, and polyphenols content and antioxidant activity. On day 1, physicochemical analysis (fat, protein, moisture, and ash content) of yogurts were determined.

#### 3.5.1. Physicochemical Analysis

The chemical composition of the yogurt samples corresponding to day 1. Moisture, ash, fat, and protein content were determined in triplicate according to the methods established by the AOAC (926.08, 935.42, 989.05, and 991.20, respectively).

#### 3.5.2. pH and Syneresis

The pH was measured in 20 g of each sample with a digital potentiometer (Orion Versa Star) previously calibrated and syneresis was evaluated using the method described by Özturk and Óner [78]; 10 g of yogurt were taken (4 ± 1 °C), placed in Corning UHP tubes (50 mL), and centrifuged (Avanti Model J-26 XPI Beckman Coulter^®^ centrifuge, USA) at 2800× *g* for 10 min, at 4 ± 1 °C. The supernatant was weighed and expressed as a percentage in relation to the initial weight of the yogurt.

#### 3.5.3. Color

Color was measured with a Model CR-410 colorimeter (Konica Minolta^®^, Japan) evaluating the L*, a* and b* parameters. Where L* is an indicator of luminosity (from black to white), a* is an indicator that goes from green (negative values) to red (positive values), and b* indicates shades from blue (negative values) to yellow (positive values). The samples were placed in plastic containers with a capacity of 200 mL. The determinations were made in triplicate, and the L*, a*, and b* values were used to calculate Chroma* (C*), Hue angle (h°), and ∆E.

The Chroma value indicates the intensity or color saturation and was determined using the following equation [62]:

(1) $$Chroma={\left(a^{*2}+b^{*2}\right)}^{1/2}$$

Hue angle refers to hues and can range from 0° (pure red), 90° (pure yellow), 180° (pure green), and 270° (pure blue). This parameter was calculated using the following equation [62]:

(2) $${Hue}^{\circ }=arctan(b^{*}+a^{*})$$

For the yogurt color difference (represented as ΔE*) in each of the treatments, the average of the readings of the parameters L*, a* and b* was used using the following equation, and the control treatment was the reference of the readings [79]:

(3) $$\Delta E=\sqrt{{\left(L_{s}-L_{c}\right)}^{2}+{\left(ah_{s}-ah_{c}\right)}^{2}+{\left(bh_{s}-bh_{c}\right)}^{2}}$$

where *L_c_*, *ah_c_,* and *bh_c_* = control parameters and *L_s_*, *ah_s_,* and *bh_s_* = parameters for the different treatments.

#### 3.5.4. Yogurt Aqueous Extract

To obtain the aqueous extract, the methodology reported by Torres-Llanez et al. [80] was applied, with the modification (or elimination) of the preparation or conditioning of the sample. Briefly, 50 mL of each treatment was placed in plastic tubes (for centrifuge) and centrifuged (Avanti^®^ J-26 XPI. Beckman Coluter^®^, USA) at 10,000× *g* for 30 min at 4 °C. The supernatant (aqueous extract) was filtered with Whatman ™ filter paper (GE Healthcare, UK) with 125mm pore diameter. Said extract was centrifuged again with the conditions mentioned above. The supernatants were filtered through a 0.22 µm pore polyethylene filters for the determination of antioxidant capacity and polyphenol content (Millipore Corp., Bedford, MA, USA). To determine betalain content, the extract was filtered on 0.45 µm pore paper (Millipore Corp., Bedford, MA, USA). All extracts were stored at −20 °C until analysis.

#### 3.5.5. Antioxidant Activity

Antioxidant activity by the ABTS (2,2′-azino-bis (3-ethylbenzothiazoline-6-sulfonic acid)) methodology was conducted according to Thaipong et al. [81]. First, an ABTS 7.4 mM solution was elaborated (38.8 mg of crystallized ammonium salt of ABTS in 10 mL of distilled water). Next, a solution 2.6 mM of potassium persulfate was prepared (6.6 mg in 10 mL with distilled water). Then, these two solutions were then combined and let to stand at room temperature for 12 h in the dark. To make the ABTS working solution, 1 mL of ABTS free radical solution was added to 60 mL of methanol to get an absorbance of 1.1 + 0.02. After that, in a 3 mL plastic cell, 150 µL of the standard (Trolox) or sample and 2850 µL of ABTS working solution were placed and let to stand for 2 h at room temperature in the dark; then, the absorbance was measured with a UV spectrophotometer at 734 nm (UV–1800. Shimadzu, Japan). Measurements were conducted three times. Antioxidant activity was given as equivalent mM Trolox (mM TE/100 g). The resulting absorbance was then put into the regression equation (y = −1.0726x + 0.9863; r^2^ = 0.9967) derived from the Trolox calibration curve.

Antioxidant activity by the DPPH (2,2-diphenyl-1-picrylhydrazyl) methodology was realized according to Thaipong et al. [81] with slight modifications. First, a 0.6 mM DPPH stock solution was elaborated (0.0240 g of DPPH in 100 mL of methanol) to get a concentration of 0.6 mM. This stock solution was kept in the dark and frozen at −20 °C until used. To obtain the working solution, 10 mL of the stock solution was mixed with methanol (45 mL) to get an absorbance of 1.1 + 0.02 and a final concentration of 0.1 mM. Later, in a 3 mL quartz cell, 150 µL of standard (Trolox) or sample and 2850 µL of the DPPH working solution were sited and let to stand in the dark at room temperature for 3 h. Finally, the absorbance was measured at 515 nm on a UV spectrophotometer (UV-1800. Shimadzu, Japan). Measurements were conducted three times. Antioxidant capacity was given as equivalent mM Trolox (mM TE/100 g). The resulting absorbance was then put into the regression equation (y = −1.3055x + 1.1077; r^2^ = 0.9994) derived from the Trolox calibration curve.

The ABTS and DPPH standard curves were executed according to Thaipong et al. [81] methodology. A stock solution (31.3 mg of Trolox (6-hydroxy-2,5,7,8-tetramethylchroman-2-carboxylic acid) in 10 mL of methanol) was made and 1.20, 1.00, 0.80, 0.60, 0.40, 0.20, 0.10, 0.08, 0.05, and 0.03 mM dilutions were used to obtain the curves.

#### 3.5.6. Polyphenols Content

The polyphenols content was determined according to Singleton and Rossi [82] with some modifications, following the Folin–Ciocalteu spectrophotometric method employing gallic acid (GA) as a standard. First, a solution of a sample extract (50 µL), distilled water (3 mL), Folin–Ciocalteu reagent (250 µL), and sodium carbonate solution at 7% (750 µL) was prepared and stirred for 10 s and let to stand at room temperature for 8 min. Next, distilled water (950 µL) was added, and the mixes were left in the dark at room temperature for 2 h. Finally, in a UV spectrophotometer (UV-1800. Shimadzu, Japan), the absorbance was taken. Triplicate measurements were taken. The absorbance measurements were linearized using the calibration curve’s regression equation (y = 0.0929x − 0.0197; r^2^ = 0.9991) and reported in mg gallic acid equivalent (mg GAE/g).

The standard curve for polyphenols content was obtained following Xu and Chang, 2007 [83] methodology. For this, a stock solution was made (0.5 g of gallic acid in 250 mL of distilled water) and 400, 300, 200, 150, 100, 80, 60, 40, and 20 ppm concentrations were prepared to elaborate the standard curve.

#### 3.5.7. Extraction of Betalains

The extraction of betalains was done according to Güneşer [84]. For this, 4 mL of the aqueous extract and 4 mL of trichloroacetic acid (TCA) solution at 4% concentration were placed and mixed in Corning tubes. Then, solutions were homogenized for 3 min with a vortex (Ultra-Turrax IKA T18 basic) and centrifuged at 4032× *g* (Avanti^®^ J-26 XPI. Beckman Coluter^®^, Indianapolis, IN, USA) for 10 min at 25 °C. Finally, using a 0.45 µm pore polyethylene filter (Millipore Corp., Bedford, MA, USA) supernatants were filtered through. Samples were stored at −20 °C for further analysis.

#### 3.5.8. Total Betalains Content

Total betalains content was determined according to Ruíz-Gutiérrez et al. [62]. McIlvaine buffer (pH 6.5, citrate-phosphate in a 1 to 10 ratio) was used to dilute the aqueous extracts of T2. This dilution was not required for T3 and T4 to produce values at their respective absorption maxima. The following formula was used to compute TB:

(4) B [mg/g ]=[(A × DF × MW × V)/(Є × L)]

where *A* = value at maximum absorption (534 for BC and 480 for BX) at 600 nm, *DF* = dilution factor, *MW* = molecular weight (550 g/mol for BC and 308 g/mol for BX), *V* = volume of the solution (1000 mL), *Є* = molar extinction coefficient (60,000 Lmol ^−1^ cm^−1^ for BC and 48,000 Lmol ^−1^ cm^−1^ for BX), and *L* = length of the reading cell (1 cm).

BC and BX quantifications were done separately, and the findings were combined to get the BT content. These determinations were done in triplicate, and the results were expressed in milligrams per 100 g of powder.

#### 3.5.9. Total Protein Concentration

The total protein concentration was evaluated following the methodology of Bradford [85]. For this, 0.1 mL of the sample and 1 mL of the Bradford reagent were mixed and let to stand in the dark for 45 min. After that, absorbance was read at 595 nm in a spectrophotometer (UV-1800 Shimadzu). The absorbance measurements were linearized using the calibration curve’s regression equation (y = 0.1526x − 0.0597; r^2^ = 0.9973) and reported in µ/mL of BSA.

For the calibration curve, bovine serum albumin (BSA) at different concentrations (1.20, 1.00, 0.80, 0.60, 0.40, 0.20, and 0.10 mM.) were prepared and the absorbance of these were linearized to obtain the regression equation of the calibration curve.

#### 3.5.10. Statistical Analysis

All analyses were conducted using SAS 9.0 program (Institute Inc., Cary, NC, USA, 2006). To determine the type, strength, and significance of their linear association, an analysis of correlations between pairs of response variables was performed, using the CORR procedure. Yogurts proximal analysis were analyzed using the ANOVA procedure, through the following model:

(5) Yij=µ+Ti+Ɛij. 

where *Yij* = response variable measured in the *j*-th repetition of the *i*-th treatment, *µ* = general mean common to all observations, *Ti* = effect of the *i*-th treatment, and *Ɛij* = random error measured in the *j*-th repetition of the *i*-th treatment, which was assumed to be identically and independently distributed in a normal way with mean *µ* and variance σ2. When there were differences across treatments, the Tukey’s and Duncan’s tests were used to perform a multiple comparison of means.

For the analysis of the physical characteristics (color, pH, and syneresis) and the bioactivities (antioxidant, betalains, and polyphenols) measured over time, an analysis was carried out with the MIXED procedure, based on the following model:

(6) Yijk=µ+Ti+Pj+Ɛijk

where *Yij* = response variable measured in the *k*-th repetition of the *i*-th treatment evaluated in the *j*-th storage time, *µ* = general mean common to all observations, *Ti* = effect of the *i*-th treatment, *Pj* = effect of j-th storage time, and *Ɛijk* = random error measured in the *j*-th repetition of the *i*-th treatment evaluated in the *j*-th storage time, which was assumed to be identically and independently distributed in a normal way with mean *µ* and variance σ2.

## 4. Conclusions

The results of this research confirm that beet extracts (liquid or lyophilized) can be added to yogurt to increase its functional properties. The addition of beet extracts significantly increased the antioxidant activity of the yogurts; where betalains and other polyphenols are the main compounds responsible for said bioactivity. Meanwhile, encapsulation of beet extract by lyophilization turned out to be an effective method to stabilize betalains. However, it was not possible to determine whether maltodextrin or inulin was better as an encapsulating agent, as the AA of the yogurt added with these varies during storage time. The mechanisms that affect antioxidant activity during fermentation are considerably varied; therefore, it is recommended to study the AA of yogurts added with liquid and/or lyophilized beet extract, with the same betalain content to obtain comparable results. The shelf life of the product could be increased to observe the behavior of betalains and polyphenols in yogurt after 14 days. In addition, it would be interesting to carry out the microbiological and economic study of this yogurt.

## Figures and Tables

**Table 1:** Physicochemical analysis of yogurt added with beet extracts (*Beta vulgaris* sp.).

Treatment	% Fat	% Protein	% Moisture	% Ashes
T1	5.09 ± 0.45 ^a^	3.87 ± 0.31 ^b^	78.94 ± 0.15 ^b^	0.18 ± 0.01 ^a^
T2	5.02 ± 0.86 ^a^	3.88 ± 0.36 ^b^	79.92 ± 0.12 ^a^	0.18 ± 0.01 ^a^
T3	5.49 ± 0.81 ^a^	4.88 ± 0.22 ^a^	78.10 ± 0.17 ^d^	0.18 ± 0.02 ^a^
T4	5.36 ± 0.32 ^a^	4.84 ± 0.48 ^a^	78.60 ± 0.33 ^c^	0.19 ± 0.02 ^a^

T1 = Natural yogurt (Y), T2 = Y added with beet juice, T3 = Y added with encapsulated extract with Maltodextrin and T4 = Y added with encapsulated extract with Inulin. ^a,b,c,d^ Values with different superscript between rows indicate significant statistical difference between treatments (*p* < 0.05).

**Table 2:** Color values L*, a*, b*, C*, h* and ∆E of yogurt added with liquid extract and lyophilized of beet (*Beta vulgaris* sp.).

Parameter	Day	T1	T2	T3	T4
L*	1	96.354 ± 2.543 ^Aa^	43.700 ± 1.376 ^Db^	79.330 ± 1.441 ^Cb^	83.678 ± 0.659 ^Ba^
	7	88.170 ± 1.294 ^Ab^	41.780 ± 1.828 ^Dc^	82.079 ± 2.189 ^Ca^	80.719 ± 0.503 ^Bc^
	14	87.528 ± 2.448 ^Ac^	50.884 ± 1.742 ^Da^	76.927 ± 2.739 ^Cc^	82.225 ± 1.033 ^Bb^
a*	1	−3.522 ± 0.124 ^Cb^	32.763 ± 1.729 ^Ab^	22.705 ± 0.998 ^Ba^	21.076 ± 0.688 ^Bb^
	7	−3.272 ± 0.232 ^Ca^	30.701 ± 1.254 ^Ab^	21.581 ± 0.601 ^Bb^	22.822 ± 0.051 ^Ba^
	14	−3.266 ± 0.106 ^Ca^	39.563 ± 3.047 ^Aa^	22.137 ± 0.816 ^Bb^	22.118 ± 0.732 ^Ba^
b*	1	8.612 ± 0.303 ^Ab^	2.174 ± 0.209 ^Bb^	0.617 ± 0.097 ^Db^	1.574 ± 0.380 ^Ca^
	7	9.533 ± 0.281 ^Aa^	3.254 ± 0.077 ^Ba^	1.344 ± 0.248 ^Da^	0.696 ± 0.024 ^Cb^
	14	9.037 ± 0.132 ^Aa^	3.527 ± 0.414 ^Ba^	1.182 ± 0.117 ^Da^	1.169 ± 0.127 ^Ca^
C*	1	9.305 ± 0.278 ^Cb^	32.835 ± 1.735 ^Ab^	22.713 ± 0.998 ^Ba^	21.135 ± 0.673 ^Bb^
	7	10.078 ± 0.314 ^Ca^	30.873 ± 1.241 ^Ab^	21.623 ± 0.595 ^Bb^	22.833 ± 0.503 ^Ba^
	14	9.609 ± 0.134 ^Cb^	39.720 ± 3.067 ^Aa^	22.169 ± 0.819 ^Bb^	22.149 ± 0.732 ^Ba^
h*	1	2.445 ± 0.134 ^Cc^	0.066 ± 0.005 ^Ac^	0.027 ± 0.004 ^Bc^	0.075 ± 0.019 ^Ba^
	7	2.913 ± 0.168 ^Ca^	0.106 ± 0.006 ^Aa^	0.062 ± 0.012 ^Ba^	0.030 ± 0.002 ^Bc^
	14	2.767 ± 0.091 ^Cb^	0.089 ± 0.006 ^Ab^	0.053 ± 0.005 ^Bb^	0.053 ± 0.006 ^Bb^
∆E	1	-	64.268 ± 1.584 ^Aa^	32.273 ± 1.803 ^Ba^	28.553 ± 1.059 ^Ca^
	7	-	57.841 ± 1.971 ^Ab^	26.867 ± 0.739 ^Bb^	28.539 ± 0.690 ^Ca^
	14	-	56.635 ± 2.469 ^Ab^	28.626 ± 1.782 ^Bb^	27.099 ± 1.012 ^Cb^

T1 = Natural yogurt (Y), T2 = Y added with beet juice, T3 = Y added with extract encapsulated with Maltodextrin and T4 = Y added with extract encapsulated with Inulin. L* = luminosity, a* = green (-) and red (+), b* = blue (-) and yellow (+), C* = chroma (color saturation), h* = Hue angle (hue), and ∆E = color difference. ^A,B,C,D^ Values in columns with different superscript indicate significant statistical difference between treatments (*p* < 0.05). ^a,b,c^ Values in rows with different superscript indicate significant statistical difference over time (*p* < 0.05).

**Table 3:** Correlation between the response variables of yogurt added with liquid extract and freeze-dried beet extracts (*Beta vulgaris* sp.).

	DPPH˙	TP	TB	BC	BX	TPC	L*	a*	b*	pH	SYN
ABTS˙	0.9652	0.8263	0.9281	0.9231	0.9361	0.8022	−0.9306	0.8322	−0.4192	0.3101	0.7143
*p*-value	<0.0001	<0.0001	<0.0001	<0.0001	<0.0001	<0.0001	<0.0001	<0.0001	<0.0001	<0.0001	<0.0001
DPPH˙		0.8002	0.9395	0.9355	0.9447	0.8128	−0.9463	0.8017	−0.3518	0.2469	0.7660
*p*-value		<0.0001	<0.0001	<0.0001	<0.0001	<0.0001	<0.0001	<0.0001	<0.0001	<0.0001	<0.0001
TP			0.8615	0.8621	0.8552	0.7917	−0.7921	0.4929	0.0126	0.2085	0.7890
*p*-value			<0.0001	<0.0001	<0.0001	<0.0001	<0.0001	<0.0001	0.8217	0.0002	<0.0001
TB				0.9994	0.9960	0.8822	−0.9410	0.7054	−0.1638	0.2006	0.8773
*p*-value				<0.0001	<0.0001	<0.0001	<0.0001	<0.0001	0.0031	0.0003	<0.0001
BC					0.9924	0.8802	−0.9355	0.6979	−0.1525	0.1949	0.8802
*p*-value					<0.0001	<0.0001	<0.0001	<0.0001	0.0059	0.0004	<0.0001
BX						0.8827	−0.9502	0.7214	−0.1927	0.2147	0.8645
*p*-value						<0.0001	<0.0001	<0.0001	0.0005	<0.0001	<0.0001
TPC							−0.8939	0.5308	−0.0260	0.2972	0.8480
*p*-value							<0.0001	<0.0001	0.6416	<0.0001	<0.0001
L*								−0.7702	0.3215	−0.2102	−0.8032
*p*-value								<0.0001	<0.0001	0.0001	<0.0001
a*									−0.7934	0.1319	0.4245
*p*-value									<0.0001	0.0175	<0.0001
b*										−0.1343	0.1679
*p*-value										0.0156	0.0024
pH											−0.0415
*p*-value											0.4562

ABTS˙ and DPPH˙ = Antioxidant activity, TP = Total polyphenols, TB = Total betalains, BC = Betacyanins, BX = Betaxanthins, TPC = Total protein concentration, L* = Luminosity, a* (tendency to red), b* (tendency to green), and SYN = Syneresis. *p*-value = Level of significance of the correlation.

## Data Availability

The data presented in this study are available on request from the corresponding author.

## Sample Availability

Samples of the compounds …… are available from the authors.
